# Physical multimorbidity and lifetime suicidal ideation and plans among rural older adults: the mediating role of psychological distress

**DOI:** 10.1186/s12888-021-03087-4

**Published:** 2021-02-06

**Authors:** Zhengyue Jing, Jie Li, Pei Pei Fu, Yi Wang, Yemin Yuan, Dan Zhao, Wenting Hao, Caiting Yu, Chengchao Zhou

**Affiliations:** 1 Centre for Health Management and Policy Research, School of Public Health, Cheeloo College of Medicine, Shandong University, 44 Wen-hua-xi Road, Jinan, 250012 Shandong China; 2 NHC Key Lab of Health Economics and Policy Research, Shandong University, 44 Wen-hua-xi Road, Jinan, 250012 Shandong China

**Keywords:** Physical multimorbidity, Psychological distress, Suicidal ideation and plans, Older adults, Rural area

## Abstract

**Background:**

Previous studies have revealed that single physical chronic condition was associated with suicidal ideation/plans, but few studies have examined the relationship between multimorbidity and suicidal ideation/plans, and no studies have explored the underlying potential mechanism on this relationship in China. This study aimed to explore association between physical multimorbidity and suicidal ideation as well as plans, and further examine the mediating role of psychological distress (PD) on this relationship.

**Methods:**

This study was based on the data from a survey about the health service of rural elderly household in Shandong, China. A total of 3242 adults aged 60 years and older were included in this study. PD was measured by Kessler Psychological Distress Scale (K10). Ordinal and binary logistic regression analyses were employed to explore the association between physical multimorbidity, PD and suicide ideation/plans. Bootstrapping analysis was further used to examine the mediation effect of PD on the association of multimorbidity and suicidal ideations/plans.

**Results:**

The prevalence of multimorbidity, lifetime suicidal ideation, and suicidal plan in rural older adults was 35.2, 10.6 and 2.2%, respectively. Older adults living in rural areas with two or more chronic physical conditions experienced significantly higher risk of suicidal ideation and suicidal plans. The association between multimorbidity and suicidal ideations/plans was partially mediated by PD, of which, the mediating effect of PD accounted for 31.7 and 25.5% of the total effect, respectively.

**Conclusion:**

This study demonstrated the associations between physical multimorbidity and suicidal ideation/plans, and the mediating role of PD on this relationship among Chinese rural elderly. Healthcare providers in rural community should provide regular surveillance for the mental health status among the rural elderly with multimorbidity, and carry out various effective intervention measures to improve the mental health status, so as to reduce the risk of suicide.

## Background

Suicide is a serious public health issue worldwide and nearly 800,000 people died from suicide every year. Although the overall suicide rate in China has decreased over the past few decades [1, 2], it remains high among the elderly in China [3]. A recent study indicated that suicide rate among the elderly aged 65–85 years was 2.75–7.08 times higher than that of the general population in China [4]. According to World Health Organization (WHO) report, the suicide rate was highest in population aged over 70 years across different regions in the world. Currently, the suicide rate is about 51.5 per 100,000 people among the elderly in China [5, 6]. This rate was found to be significantly higher among older people in rural than that in urban areas [7, 8]. A study revealed that the mean annual suicide rate among the elderly aged 60–84 years in rural and urban areas was 82.8 per 100,000 people and 16.7 per 100,000 people, respectively [9]. With a rapid increase of elderly population, there is an urgent need to prevent suicide among the older adults, especially in rural areas.

Previous studies have shown that suicidal ideation, plans, and attempts were important predicators of later suicide [10–13]. The early detection of suicidal ideation and plans is one of the critical primary prevention measures for suicide [14]. However, the current prevention measures mainly focus on the secondary prevention (suicide attempts) and the tertiary prevention (suicide) [15, 16]. Therefore, to identify potential influencing factors for suicidal ideation and plans is of priority.

Physical chronic conditions were found to be related with higher odds of reporting suicidal behaviors among the elderly [17, 18]. Moreover, more and more people were found to be affected by more than one physical condition, especially among the elderly [19, 20]. The existence of two or more physical chronic conditions in an individual was known as multimorbidity [19]. Previous studies in some other countries further found that multimorbidity was associated with suicidal related behaviors. A study by Scott and colleagues revealed that the risk of suicidal outcomes was increased with the increase of the number of physical chronic conditions [21]. Another study in Korea showed that the adults with multimorbidity experienced higher prevalence of suicide thoughts and plans [22]. Although previous studies have demonstrated that the relationship between multimorbidity and suicidal ideation/plans, few studies have explored this association among the elderly, and the underlying potential mechanism or pathways on this relationship is still rarely explored.

Psychological distress (PD) is largely defined as a state of emotional suffering, typically characterized by depressive and anxiety symptoms. PD is an important and widely-used indicator in evaluating the psychological health of the population [23–25], which was assessed by Kessler Psychological Distress Scale (K10) in the present study. The relationship between single chronic conditions (such as cancer, diabetes, hypertension) and mental disorder has been well established [26]. Recently, more and more studies have focused on the relationship between multimorbidity and PD. A study conducted in Australia showed that the risk of PD increased with the number of chronic conditions in adults [27]. Another study further showed that three or more chronic conditions conferred a 2.30-fold increase in elevated anxiety among the older adults [28]. Furthermore, a number of studies have examined the association between PD and suicidal related behaviors and revealed that PD was correlated with suicidal ideation, plans and attempts [29, 30]. Similarly, PD was also found to be the strongest predicator of suicidal ideation across different age groups [31]. About 90% of suicides were found to have mental disorders before their death [32]. Thus, PD might be a mediator between the association between chronic conditions and suicidal ideations or plans.

The aim of the present study is to investigate the prevalence of suicidal ideations/plans and further examine the association between physical multimorbidity and suicidal ideation and suicidal plans, as well as to explore the mediating role of PD on this relationship among rural elderly in China. The main hypotheses of this study are as follows: First, there is a significant association between physical multimorbidity and suicidal ideations as well as suicidal plans in rural older adults; Second, PD is a mediator between multimorbidity and suicidal ideations as well as suicidal plans in rural older adults.

## Methods

### Data source and sample

Data of this study were from a survey about the health status of rural elderly in Shandong, China, which was conducted in 2019. Shandong is the second most populous province of China with a number of 107 million people in 2018, and also the province with largest number of elderly population in China.

This study used a three-stage stratified cluster method to select the participants, which was explained in detail elsewhere [33]. Finally, 3242 elderly living in the study sites were interviewed, with a respondent rate of 90.05%.

### Measurements

#### Lifetime suicidal ideation/plans

In this study, suicidal ideation was measured by a widely used question of “Have you ever seriously considered about suicide/killing yourselves?” For those who answered “Yes”, they would be further asked “Have you ever make a plan for suicide?” to measure suicidal plans. This question is from the US National Comorbidity Survey (NCS) [34], and many previous studies conducted in China also used this question to measure suicidal ideation, which was proved to be of high reliability and validity [35].

#### Physical multimorbidity

Physical chronic condition was measured by using the self-reported question that “Have you ever been diagnosed with a chronic condition by a physician?” The answer contained “Yes” or “No”, and if the answer was “Yes”, they would be further asked “How many chronic conditions have you ever been diagnosed by a doctor?” The chronic conditions in this study only refer to physical chronic illness. In order to ensure the accuracy of the interview information, the interviewers would further ask the interviewees about their medication details and also sought help from the village doctors to validate the physical chronic conditions information in the health management system in the sampling villages. One person with two or more chronic conditions was defined as physical multimorbidity. This study used the ordinal forms of multimorbidity (0, 1, 2, 3, 4+) to better show the relationship between chronic conditions and other main variables.

#### Psychological distress

PD was measured by K10, which was used to evaluate the respondents’ psychological conditions that characterized by depression and anxiety [25]. Previous study in China has demonstrated the reliability and validity of the K10 [36]. This scale scored from 10 to 50 points, and the lower scores reflected the better psychological health status. Based on the nature of data, the PD was divided into four categories: low (scores range from 10 to 15), moderate (scores range from 16 to 21), high (scores range from 22 to 29), and very high PD (scores range from 30 to 50).

#### Other controlling variables

Sleep disturbance was measured by the Pittsburgh sleep quality index (PSQI) [37]. The PSQI was the self-reported questionnaire that used to assess the sleep quality/disturbance during the 1-month period, which included seven components that scoring from 0 to 21 and higher scores reflect the worse sleep quality. In this study, PSQI was dichotomized into without sleep disturbance and with sleep disturbance; the cut-off of PSIQ of 7 has received good sensitivity and specificity in China [38]. Household income was classified into four categories according to quartile, including Q1, Q2, Q3, and Q4. Among them, Q1, Q2, Q3, and Q4 represented the lowest, middle-low, middle-high, and highest economic status group, respectively. Marital status was categorized into single and married, of which, singles included the never married, the widowed, and the divorced, and married included the first marriage and re-marriage. In addition, the controlling variables also included gender, age, education, alcohol drinking, cigarette smoking, and physical exercise.

### Statistical analysis

IBM SPSS version 22.0 and Stata 14.0 were employed for analysis. The present study described the basic characteristics of respondents with mean (standard deviation) or frequencies (percentage). According to the mediation testing technology proposed by Baron and Kenny [39], the ordinal logistic regression model was firstly performed to examine the relationship between physical multimorbidity and PD. Secondly, the binary logistic regression model was performed to explore the association between physical multimorbidity and suicidal ideation as well as suicidal plans. Thirdly, the binary logistic regression model was used to further explore the association between physical multimorbidity and suicidal ideation as well as suicidal plan when PD included in this model. The statistically significant threshold was based on two-sided and 0.05-level tests. Moreover, spearman coefficient was used to determine the correlation between main variables, and nonparametric bootstrapping (with 5000 bootstrap samples) was employed to validate the mediation effect, and if the 95% CI excluded zero was regarded as statistically significant.

## Results

### Socio-demographic characteristics

The basic characteristics of the respondents were presented in Table 1. Among the 3242 participants, the majority of them were female (63.5%), with the education level of primary school or below (80.5%), married (74.5%), and had sleep disturbance (47.4%). The average age was 70.1 ± 6.2 years.

**Table 1:** Basic characteristics of the older adults (60+) in rural areas of Shandong province, China, 2019

Characteristics	N	Frequency (%)
**Total**	3242	100.0
**Multimorbidity (chronic conditions)**		
None	896	27.6
One	1205	37.2
Two	801	24.7
Three	285	8.8
Four and above	55	1.7
**Suicidal ideation**		
No	2899	89.4
Yes	343	10.6
**Suicidal plans**		
No	3172	97.8
Yes	70	2.2
**Psychological distress (level)**		
Low	1845	56.9
Moderate	637	19.6
High	515	15.9
Very high	245	7.6
**Sex**		
Male	1182	36.5
Female	2060	63.5
**Age (Years),** Mean ± SD	3242	70.1 ± 6.2
**Educational attainment**		
Illiterate	1353	41.7
Primary school	1257	38.8
Middle school or above	632	19.5
**Marital status**		
Single	827	25.5
Married	2415	74.5
**Household income**		
Q1(the poorest)	811	25.0
Q2	810	25.0
Q3	813	25.1
Q4 (the richest)	808	24.9
**Cigarette smoking**		
Never smoker	2239	69.0
Current smoker	678	20.9
Former smoker	325	10.1
**Alcohol drinking**		
Never-drinker	2320	71.5
Former drinker	207	6.4
Current drinker	715	22.1
**Physical exercise**		
No	1579	48.7
Yes	1663	51.3
**Sleep disturbances**		
No	1706	52.6
Yes	1536	47.4

### Description of physical multimorbidity, PD and suicidal ideation/plans

Regarding the suicidal ideation/plans, of the respondents, 10.6 and 2.2% reported lifetime suicidal ideation and suicidal plans, respectively. As for the chronic conditions, 35.2% of participants had more than two chronic physical illnesses, with 24.7% reporting two illnesses, 8.8% reporting three illnesses, and 1.7% reporting four and above illnesses. In the aspect of PD, 23.5% of respondents experienced high/very high level of PD.

### Association between physical multimorbidity and psychological distress

As shown in Table 2, after adjusting for sex, age, education, marital status, alcohol drinking, household income, cigarette smoking, physical exercise, and sleep disturbance, chronic conditions were significantly related with PD. The odds of reporting PD increased with the number of chronic conditions.

**Table 2:** The association between physical multimorbidity and psychological distress among the older adults (60+) in rural areas of Shandong province, China, 2019

Characteristics	Psychological distress		
	OR	***P***-value	95%CI
**Multimorbidity (chronic conditions)**			
None	1.0		
One	1.38	0.001	1.15–1.67
Two	2.05	< 0.001	1.68–2.51
Three	2.07	< 0.001	1.59–2.70
Four and above	2.37	0.001	1.40–3.99
**Sex**			
Male	1.0		
Female	1.40	0.001	1.14–1.72
**Age (Years),** Mean ± SD	0.99	0.118	0.98–1.00
**Educational attainment**			
Illiterate	1.0		
Primary school	1.07	0.370	0.92–1.26
Middle school or above	1.02	0.884	0.82–1.25
**Marital status**			
Single	1.0		
Married	0.99	0.936	0.83–1.18
**Household income**			
Q1(the poorest)	1.0		
Q2	0.84	0.083	0.68–1.02
Q3	0.82	0.071	0.67–1.02
Q4 (the richest)	0.66	< 0.001	0.53–0.81
**Cigarette smoking**			
Never smoker	1.0		
Current smoker	1.33	0.007	1.08–1.65
Former smoker	0.92	0.581	0.68–1.24
**Alcohol drinking**			
Never-drinker	1.0		
Former drinker	0.84	0.332	0.59–1.19
Current drinker	0.82	0.075	0.66–1.02
**Physical exercise**			
No	1.0		
Yes	0.69	< 0.001	0.60–0.79
**Sleep disturbances**			
No	1.0		
Yes	3.47	< 0.001	3.00–4.02

PD mediated the association between physical multimorbidity and suicidal ideation/plans. As shown in Tables 3 and 4, when PD was included in model 2, the result showed that the increasing number of chronic conditions was still significantly correlated with suicidal ideations as well as suicidal plans. Moreover, bootstrap results showed that the direct effect, indirect effect, and total effect were also statistically significant, and the total effect of physical multimorbidity on suicidal ideation as well as suicidal plans were partially explained by PD, of which, the indirect effect accounting for 31.7 and 25.5% of total effect, respectively.

**Table 3:** The mediating role of psychological distress on the association between physical multimorbidity and suicidal ideation among the older adults (60+) in rural areas of Shandong province, China, 2019

Characteristics	Suicidal ideation					
	Without mediators (Model 1)			With mediators (Model 2)		
	OR	P-value	95%CI	OR	P-value	95%CI
**Multimorbidity (chronic conditions)**						
None	1.0			1.0		
One	1.51	0.020	1.07–2.14	1.34	0.106	0.94–1.93
Two	1.94	< 0.001	1.35–2.77	1.53	0.026	1.05–2.23
Three	2.78	< 0.001	1.82–4.25	2.29	< 0.001	1.46–3.59
Four and above	4.08	< 0.001	2.04–8.19	3.29	0.002	1.55–6.99
**Psychological distress (level)**						
Low				1.0		
Moderate				2.48	< 0.001	1.73–3.56
High				4.98	< 0.001	3.55–7.00
Very high				13.70	< 0.001	9.37–20.02
**Sex**						
Male	1.0			1.0		
Female	1.62	0.010	1.12–2.33	1.47	0.048	1.00–2.15
**Age (Years)**	0.98	0.037	0.96–1.00	0.98	0.078	0.96–1.00
**Educational attainment**						
Illiterate	1.0			1.0		
Primary school	0.96	0.774	0.74–1.25	0.92	0.551	0.69–1.21
Middle school or above	0.96	0.816	0.67–1.36	0.91	0.630	0.63–1.32
**Marital status**						
Single	1.0			1.0		
Married	1.20	0.232	0.89–1.61	1.16	0.338	0.85–1.59
**Household income**						
Q1 (the poorest)	1.0			1.0		
Q2	0.77	0.127	0.56–1.08	0.83	0.296	0.59–1.18
Q3	0.72	0.060	0.52–1.01	0.80	0.224	0.56–1.14
Q4 (the richest)	0.64	0.012	0.45–0.91	0.76	0.153	0.53–1.10
**Cigarette smoking**						
Never smoker	1.0			1.0		
Current smoker	0.79	0.249	0.54–1.17	0.64	0.037	0.42–0.97
Former smoker	0.84	0.277	0.50–1.39	0.81	0.441	0.47–1.38
**Alcohol drinking**						
Never-drinker	1.0			1.0		
Former drinker	1.29	0.392	0.72–2.31	1.54	0.164	0.84–2.85
Current drinker	0.97	0.888	0.65–1.45	1.16	0.479	0.76–1.78
**Physical exercise**						
No	1.0			1.0		
Yes	0.62	< 0.001	0.48–0.79	0.61	< 0.001	0.47–0.78
**Sleep disturbances**						
No	1.0			1.0		
Yes	2.28	< 0.001	1.77–2.94	1.28	0.081	0.97–1.69

**Table 4:** The mediating role of psychological distress on the association between physical multimorbidity and suicidal plan among the older adults (60+) in rural areas of Shandong province, China, 2019

Characteristics	Suicidal plan					
	Without mediators (Model 1)			With mediators (Model 2)		
	OR	P-value	95%CI	OR	P-value	95%CI
**Multimorbidity (chronic conditions)**						
None	1.0			1.0		
One	3.17	0.020	1.19–8.39	2.78	0.042	1.04–7.45
Two	3.79	0.008	1.41–10.21	2.99	0.033	1.09–8.20
Three	5.89	0.001	2.03–17.07	4.65	0.006	1.56–13.86
Four and above	12.45	< 0.001	3.36–46.15	8.95	0.002	2.27–35.30
**Psychological distress (level)**						
Low				1.0		
Moderate				2.62	0.037	1.06–6.48
High				4.54	< 0.001	1.95–10.59
Very high				18.51	< 0.001	8.19–41.78
**Sex**						
Male	1.0			1.0		
Female	3.56	0.034	1.07–6.09	2.35	0.060	0.96–5.71
**Age (Years)**	0.96	0.088	0.92–1.01	0.96	0.132	0.92–1.01
**Educational attainment**						
Illiterate	1.0			1.0		
Primary school	0.88	0.657	0.51–1.53	0.82	0.506	0.48–1.45
Middle school or above	1.01	0.983	0.49–2.07	0.92	0.818	0.43–1.93
**Marital status**						
Single	1.0			1.0		
Married	1.53	0.194	0.80–2.93	1.46	0.264	0.75–2.84
**Household income**						
Q1(the poorest)	1.0			1.0		
Q2	0.65	0.227	0.33–1.30	0.68	0.293	0.33–1.39
Q3	0.54	0.088	0.26–1.09	0.65	0.243	0.31–1.34
Q4 (the richest)	0.68	0.278	0.34–1.36	0.84	0.636	0.41–1.72
**Cigarette smoking**						
Never smoker	1.0			1.0		
Current smoker	0.84	0.694	0.35–2.02	0.59	0.256	0.24–1.46
Former smoker	0.98	0.971	0.33–2.93	0.86	0.799	0.28–2.67
**Alcohol drinking**						
Never-drinker	1.0			1.0		
Former drinker	1.21	0.781	0.31–4.76	1.71	0.453	0.42–6.95
Current drinker	1.52	0.329	0.65–3.53	2.30	0.066	0.94–5.60
**Physical exercise**						
No	1.0			1.0		
Yes	0.45	0.002	0.27–0.75	0.52	0.018	0.31–0.89
**Sleep disturbances**						
No	1.0			1.0		
Yes	3.45	< 0.001	1.84–6.44	1.69	0.119	0.87–3.31

## Discussion

This population-based study found that physical multimorbidity was associated with lifetime suicidal ideation as well as suicidal plans in rural older adults. For the first time, this study tried to explore the potential mediating effect of PD on this relationship, and the results showed that PD was associated with suicidal ideation as well as suicidal plans, and partially mediated the association between multimorbidity and suicidal ideation/plans.

The present study found the prevalence of lifetime suicidal ideation and suicidal plans in rural elderly (60+) was 10.6 and 2.2%, respectively. The prevalence of suicidal ideation in present study was much higher than the 4.8% among rural elderly (65+) in Beijing reported by Ma et al. [40], the 5.2% in rural older adults (65+) in Shandong reported by Ge et al. [41], and the 9.4% among U.S. Chinese elderly (60+) reported by Dong and colleagues [42]. Meanwhile, the prevalence of suicidal plans in this study was higher than the 0.51% among elderly (65+) in Spain reported by Miret and colleagues. The lifetime prevalence of suicidal ideation and suicide plans in present study were both lower than 34.5 and 10.3% of elderly (65+) in a remote rural area of China that found by Chiu et al. [11]. One possible reason for such difference is that the sample size of Chiu et al.’ s study was relatively small and only 87 participants were included in the study. Another possible reason is that, the prevalence of suicidal ideation/plans was age-related. The older the elderly, the higher the prevalence. The age inclusion criteria by Chiu et al.’ s study was 65 years and above, which was older than the current study.

Congruent with our hypothesis, we found that the presence of two or more physical chronic conditions were significantly associated with suicidal ideation and suicidal plans. In the fully adjusted model, the elderly with two or more chronic conditions had higher odds of experiencing suicidal ideation and plans compared to those without any physical condition. This finding was consistent with a previous study, which demonstrated that the increasing number of chronic conditions were related with higher risks of suicidal ideation and plans in US population [43]. Huh et al. reported elderly (65+) with multimorbidity had higher prevalence of suicidal thoughts than those without multimorbidity in Korea [22]. Some previous studies showed that compared with those with no physical conditions, the elderly with multimorbidity were more likely to have disability, poor physical and mental health-related quality of life [44, 45], and it is intolerable for some older adults when these adverse consequences have accumulated to a certain extent. That was to say, multimorbidity may cause more seriously adverse health outcomes, thus increased the likelihood of suicidal ideation/plans of the patients.

Furthermore, we found that PD were independently associated with both multimorbidity and suicidal ideation/plans, and partially mediated the association between them. Although there was no study to explore the mediating role of PD in the association between multimorbidity and suicidal ideation/plans, a previous study conducted in China suggested that depression partly mediated the association between multimorbidity and health-related quality of life in older adults [46]. As mentioned above, the possible interpretation of this finding might be that the multiple adverse outcomes caused by multimorbidity may directly or indirectly increase the risk of PD, which in turn further stimulates the occurrence of the suicidal ideation/plans. A review suggested that the patients with multimorbidity were twice as likely to experience depression than those without multimorbidity [47]. A study conducted in Canada indicated that people with obesity and multimorbidity had higher probability of reporting mental disorders [48]. In addition, multimorbidity was associated with increased risk of loneliness, social exclusion, function limitations, and economic burden, all of which may negatively affect the PD [49–51]. Several previous studies also found that PD was one of the strongest influencing factors of suicidal related behaviors [52, 53]. Thus, multimorbidity may have indirect effect on suicidal ideation as well as suicidal plans via inducing the PD. Some previous studies demonstrated that health care utilization and spending increased with the number of chronic conditions among the elderly [54–56], and a large proportion of the healthcare cost for multimorbidity was out-of-pocket paid by the rural elderly themselves, which may be more economically disadvantaged and impose financial hardship for their households. Moreover, as the origin of the Confucius culture, the elderly in rural Shandong were more traditional and conservative [57]. For those with multiple chronic conditions, they were fear of becoming a burden to their families and bearing higher level of PD for a long time. This existence of mental disorders may be one of the triggers of stressful life events, and ultimately increase the risk of suicide [58].

The findings in this study implied for the healthcare providers in rural communities to strengthen the regular screening and management of chronic conditions among the elderly to prevent or retard the presence of the multimorbidity, and further to reduce the chronic condition burden. More importantly, a regular surveillance for the mental health status should be taken among the rural elderly with multimorbidity, and various effective intervention measures should be carried out to improve the mental health status, so as to reduce the risk of suicide.

This study had several limitations. First, the information about number of physical chronic conditions and suicidal ideation/plans was self-reported, which might lead to recall basis. Second, although the US National Comorbidity Survey (NCS) and many previous studies have used the question “Have you ever seriously considered about suicide/killing yourselves?” to measure suicidal ideation, this question cannot be used as a standard instrument for assessing suicidal ideation. In addition, although previous studies have found that older adults who committed suicide were more likely to have antidepressants detected in the blood [59], the information about diagnosed mental illness and medication (e.g., antidepressant use information) was not collected in our study, which would be remedied in the follow-up studies. Third, based on the availability of data, we only examined the relationship between the number of chronic conditions and suicidal ideation/plans, further studies would investigate whether the pattern, length, or severity of chronic conditions were associated with suicidal ideation/plans. Fourth, the present study was based on cross-sectional design, so we could not infer the casual relationship between multimorbidity, PD, and suicidal ideation as well as plans, prospective studies were needed in the future to elucidate the causal association.

## Conclusions

This study demonstrated the significant associations between physical multimorbidity and suicidal ideation/plans, and the mediating role of PD on this relationship among Chinese rural elderly. In rural areas, policy makers and healthcare providers should strengthen the prevention and management of chronic conditions among the elderly, to prevent or retard the presence of chronic conditions, especially the multimorbidity. In addition, a regular surveillance for the mental health status should be taken among the rural elderly with multimorbidity, and various effective intervention measures should be carried out to improve the mental health status, so as to reduce the risk of suicide.

## Data Availability

Our data may not be shared directly, because it is our teamwork. Informed consent should be attained from all the team members. The datasets used and/or analyzed during the current study are available from the corresponding author on reasonable request.

## Abbreviations

PD: Psychological distress
WHO: World Health Organization
PSQI: Pittsburgh sleep quality index
